# The Role of Changes in the Expression of Inflammation-Associated Genes in the Variants of Cerebral Small Vessel Disease

**DOI:** 10.3390/ijms25158113

**Published:** 2024-07-25

**Authors:** Larisa A. Dobrynina, Angelina G. Makarova, Alla A. Shabalina, Anastasiia G. Burmak, Polina S. Shlapakova, Kamila V. Shamtieva, Maria M. Tsypushtanova, Elena I. Kremneva, Maryam R. Zabitova, Alexey S. Filatov, Elena V. Gnedovskaya

**Affiliations:** Research Center of Neurology, 80 Volokolamskoe Shosse, 125367 Moscow, Russia; dobrla@mail.ru (L.A.D.); angelinagm@mail.ru (A.G.M.); ashabalina@yandex.ru (A.A.S.); burmak_n@mail.ru (A.G.B.); shlapakovaps@gmail.com (P.S.S.); kamila.shamt@gmail.com (K.V.S.); tzipushtanova@mail.ru (M.M.T.); moomin10j@mail.ru (E.I.K.); fil4tovmd@gmail.com (A.S.F.); gnedovskaya@mail.ru (E.V.G.)

**Keywords:** cerebral small vessel disease, MRI types, white matter hyperintensity, differential gene expression, direct digital RNA detection method, inflammation-associated genes, Alzheimer’s disease

## Abstract

Age-dependent cerebral small vessel disease (CSVD) is a common disease with a high social burden characterized by heterogeneity of forms and frequent comorbidity with Alzheimer’s disease (AD). Previously, we identified two MRI types of CSVD with specific clinical presentation and, probably, different mechanisms. The present study included 34 patients with CSVD and white matter hyperintensity (WMH) of stage Fazekas (F) 3 (mean age 61.7 ± 8.9) and 11 volunteers (mean age 57.3 ± 9.7). Total RNA was isolated from peripheral blood leukocytes. The expression of 58 protein-coding genes associated with CSVD and/or AD and 4 reference genes were assessed as part of the original panel for the NanoString nCounter analyzer. Testing results were validated by real-time PCR. There was a significant decrease in the expression levels of the *ACOX1*, *CD33*, *CD2AP*, *TNFR1*, and *VEGFC* genes in MRI type 2 relative to the control group as well as a decrease in the expression level of the *CD33* gene in MRI type 2 compared to MRI type 1. Processes associated with inflammatory pathways with decreased expression of the identified genes are important in the development of MRI type 2 of CSVD. Given the direct connection of the established genes with AD, the importance of this form of CSVD in comorbidity with AD has been assumed.

## 1. Introduction

Age-dependent cerebral small vessel disease (CSVD) is a significant cause of ischemic and hemorrhagic strokes, cognitive impairment (CI), and gait disorders and pelvic organ dysfunction as well as a leading modifiable risk factor for Alzheimer’s disease (AD) [1,2,3,4,5,6,7]. The prevalence of CSVD is extremely high, and neuroimaging manifestations of the disease (white matter hyperintensity (WMH), small subcortical infarcts, lacunes, cerebral microbleeds, enlarged perivascular spaces, and cerebral atrophy) are present in almost every person over the age of 60 years [8]. In most patients, CI is the earliest sign of CSVD. However, the severity and profile of CI significantly differs among patients matched by gender and age with similar MRI findings [9]. However, the severity of arterial hypertension (AH), the main risk factor for CSVD, has no direct causal relationships with cerebral MRI findings and the severity of CI [10,11,12], and its management does not slow down the progression of the disease in a significant number of cases [11,12,13]. The complex interdependencies of clinical manifestations with diagnostic MRI signs indicate the heterogeneity of CSVD forms [14,15,16,17]. This may be due to differences in the predominance of pathogenetic mechanisms of the disease, of which ischemia/hypoxia, high permeability of the blood–brain barrier (BBB), and inflammation are leading ones [5,18].

To identify different forms of age-dependent CSVD, we previously evaluated each of the diagnostic MRI signs according to a four-point severity system in different cerebral lobes and three sections of the white matter in 96 patients with CSVD with WMH Fazekas grade 3 [19]. The obtained data on the localization and severity of diagnostic MRI signs of CSVD were used to evaluate their classification options (hierarchical agglomerative method and iterative k-means algorithm) [19]. Two MRI types of CSVD were established. They differed by combination and location of MRI signs, severity of clinical symptoms, age, gender, and blood markers [19]. In MRI type 1, patients were significantly younger, without gender predominance, and had more pronounced CI and gait disorders. In MRI type 2, patients were older, with male predominance. The course of MRI type 1 disease was associated with a decrease in the blood level of vascular endothelial growth factor A (VEGF-A) and MRI type 2 with an increase in the blood level of tumor necrosis factor–α (TNF–α) [19]. The data obtained suggested the presence of different pathogenetic variants of CSVD and the possibility of their diagnosis by MRI [19].

The study of CSVD heterogeneity, based on blood markers associated with potential mechanisms of disease development (endothelial dysfunction, high BBB permeability, and inflammation), has promise for clarifying the forms of the disease. In this regard, it is of particular interest to study the role of protein-coding genes associated with these processes in CSVD development, established through repeated genome-wide association studies (GWAS). In the present study, to create an original gene expression panel, we selected genes identified from repeated GWAS in CSVD, AH, and AD as well as genes encoding proteins for inflammation and BBB damage that have been demonstrated to play a role in CSVD pathogenesis. The predominance of mixed vascular-degenerative forms of CI in the population became the basis for including genes, which are significant in AD development, in the novel panel [20,21]. In addition, we assumed that previously established types of CSVD may have differences in the risks of developing comorbidity with neurodegenerative diseases. Differentiation of CSVD forms, the discovery of common mechanisms significant for CSVD and AD, and identification of potential biomarkers for the development of their comorbidity will make it possible to predict the course of the disease and the severity of its clinical symptoms.

In this study, the differential expression of protein-coding inflammation-associated genes was analyzed as part of the inventive panel using direct digital RNA detection on the NanoString nCounter platform to expand understanding of the molecular genetic mechanisms of CSVD and its heterogeneous forms (MRI types).

The aim of the study was to evaluate the expression profile of inflammation-associated genes in CSVD and its various MRI types.

## 2. Results

### 2.1. Clinical and Neuroimaging Characteristics of Two MRI Types of CSVD

Initially, CSVD patients and controls did not differ statistically in gender and age. The comparative characteristics of risk factors and clinical and neuroimaging manifestations of patients with two F3-grade MRI types and controls are presented in Table 1.

The two MRI types differed significantly by age, as MRI type 1 patients were younger (mean age 56.4 ± 8.1 vs. 67.1 ± 6.3) and included more females (58.8%), while males were prevalent (88.2%) in MRI type 2. Both types did not differ by the presence and severity of the main vascular risk factors. MRI type 1 patients had more pronounced CI and a lower MoCA score of 21 [17; 23] versus 24 [21; 26] in MRI type 2 patients as well as impairment of executive functions and memory. Lacunes and microbleeds in various parts of the brain were more prevalent in patients with MRI type 1.

### 2.2. Expression of Inflammation-Associated Genes: CSVD Group vs. Control and MRI Types 1 and 2 CSVD vs. Control

**Figure 1:**
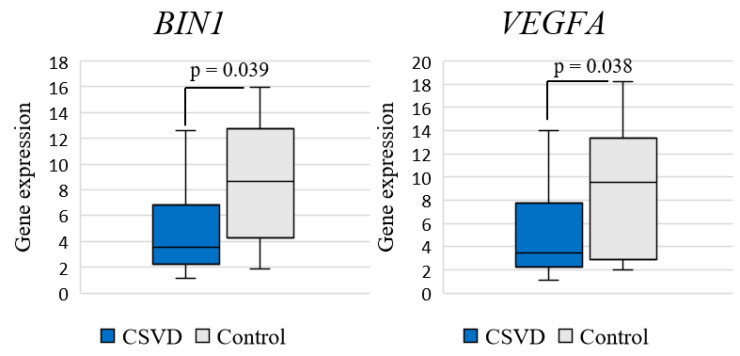
Comparison of gene expression between CSVD and controls.

Analysis of differential gene expression in the entire CSVD group compared to the control group revealed a significant decrease in the expression of *BIN1* (log2FC = −1.272; *p* = 0.039) and *VEGFA* (log2FC = −1.441; *p* = 0.038) genes (Figure 1).

**Figure 2:**
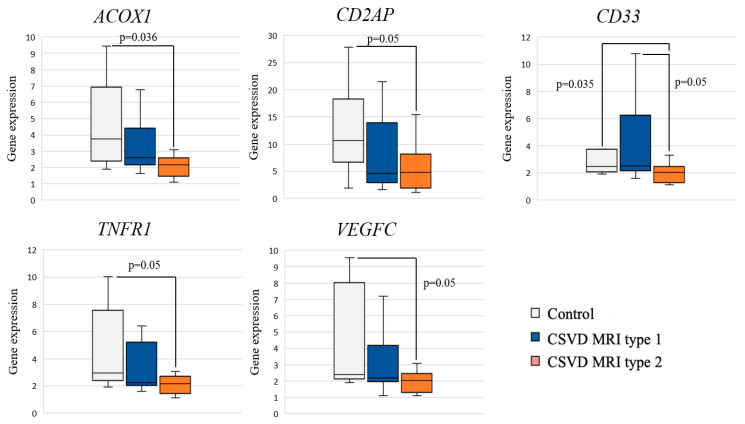
Comparison of gene expression between MRI types and controls.

Analysis of the differential gene expression in the CSVD group with MRI type 2 compared to the control group revealed a significant decrease in the expression of *ACOX1*, *CD33*, *CD2AP*, *TNFR1*, and *VEGFC* in the CSVD group (Figure 2).

Pairwise intra-group comparisons showed that gene expression in MRI type 1 had no significant differences with the control group. A significant decrease in *CD33* gene expression was found in MRI type 2 compared to MRI type 1 and control.

**Figure 3:**
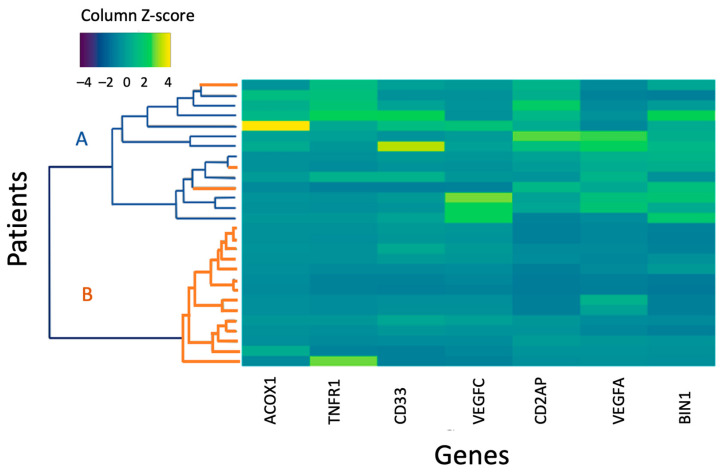
A heat map of the normalized gene expression level of MRI type 2 and control group patients. A is the control group; B is the main group. An increase in the intensity of the blue scale reflects a decrease in the level of expression, while the yellow scale reflects an increase in the level of expression.

Genes whose expression showed significant differences between the entire CSVD group and the control group, MRI types 1 and 2 and the control in comparative analysis were used to perform a hierarchical cluster analysis in MRI type 2 patients and the control group (Figure 3).

The first cluster included participants of the control group and three MRI type 2 patients with a higher level of gene expression according to the color scale; the second cluster combined MRI type 2 patients with reduced gene expression.

### 2.3. Comparative Analysis of Gene Expression with Cognitive Impairment

A comparative analysis of gene expression in MRI type 1 CSVD patients with clinically significant CI (mild CI (MCI) and dementia) and the control group showed a decrease in the *VEGFA* gene (*p* = 0.034) in patients with CI.

A comparative analysis of gene expression in MRI type 2 CSVD patients with clinically significant CI (MCI and dementia) and the control group showed a decrease in the *ACOX1*, *BIN1*, *CD2AP*, *CD33*, *PSEL*, *TNFR1*, *VEGFA*, and *VEGFC* genes (*p* < 0.05) in patients with CI.

**Figure 4:**
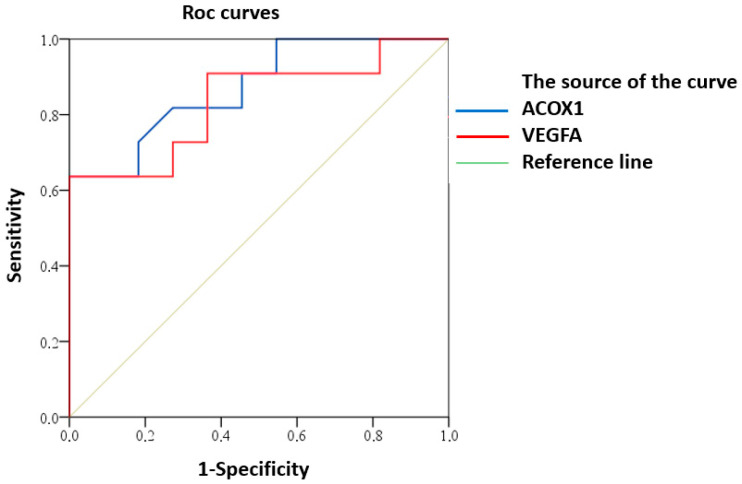
ROC curve of predictive value for clinically significant CI in MRI type 2 CSVD with reduced expression of *ACOX1* and *VEGFA*.

ROC analysis demonstrated predictive potential of reduced expression of *ACOX1* and *VEGFA* for clinically significant CI in MRI type 2 CSVD patients (Figure 4).

Good predictive ability was demonstrated for both *ACOX1* (AUC (95% CI) 0.87 (0.73–1.00), *p* = 0.003) and *VEGFA* (AUC (95% CI) 0.84 (0.66–1.00), *p* = 0.008).

The results obtained on the NanoString nCounter platform were confirmed by real-time PCR. The mRNA expression of the tested genes by real-time PCR was also reduced in the main group compared to the control group: *ACOX1* (*p* = 0.021), *BIN1* (*p* = 0.013), *CD2AP* (*p* = 0.019), *TNFR1* (*p* = 0.026), and *VEGFA* (*p* = 0.011).

## 3. Materials and Methods

### 3.1. Selection of Study Participants

The study included patients from the database of patients with CSVD who underwent inpatient treatment at the Research Center of Neurology for the period from 2016 to 2022 and had a complete MRI examination protocol. Since 2020, patients from this database have been recruited to participate in the study.

The study and its protocol were approved by the local Ethics Committee of the Research Center of Neurology (Protocol No. 10-6/20 dated 27 November 2020).

Inclusion criteria: patients aged 46–75 years with cognitive complaints and MRI brain findings that meet the STRIVE criteria [22] with T2/FLAIR Fazekas grade 3 (F3) WMH, who signed voluntary informed consent to participate in the study and for personal data processing.

Exclusion criteria: atherosclerosis with intra-/extracranial artery stenosis >50%; lobar and deep intracerebral hemorrhages or superficial hemosiderosis; inflammatory and infectious changes in cerebrospinal fluid (CSF) or CSF biomarkers of Alzheimer’s disease; acute and subacute period of small subcortical infarction (up to 3 months); acute infection, including COVID-19, or exacerbation of a chronic disease 3 months before blood sampling; decompensated general medical and endocrine pathology.

**Figure 5:**
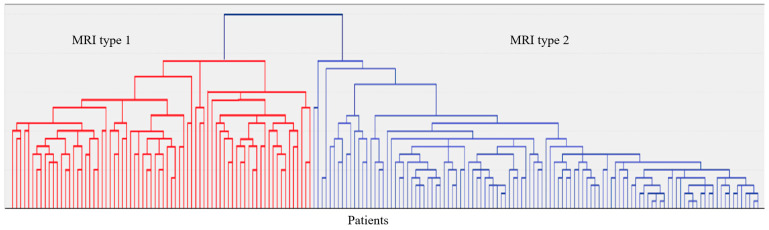
A dendrogram of the hierarchical classification of 187 patients with CSVD with the allocation of two large clusters—MRI type 1 and MRI type 2.

In 187 patients, brain MRI (Siemens Magnetom Prisma 3 Tl (Siemens AG, München, Germany) was available, including the following sequences: T2-weighted, 3D T1-mpr, 3D FLAIR, DWI, and SWI. Visual assessment of MRI images and qualitative analysis of MRI signs of CSVD in accordance with the STRIVE criteria [22] and analysis of MRI signs by localization and severity in a four-point system [23,24] were performed. The latter were used to evaluate the grouping of CSVD MRI signs by localization and severity (hierarchical agglomerative method and iterative k-means algorithm) into MRI types (Figure 5). Neuroimaging and clinical and laboratory features of the identified two MRI types of Fazekas grade 3 WMH are given in Appendix A (Table A1).

Thirty-four patients with CSVD and WMH grade F3 were selected for molecular genetic testing (MRI type 1: 17 patients; MRI type 2: 17 patients). The control group consisted of 11 volunteers (mean age 57.3 ± 9.7; women—63.6%) matched by age and gender without clinical and MRI signs of vascular and degenerative brain disorders.

### 3.2. Clinical and Neuroimaging Examination

Classical vascular risk factors, general cognitive level assessed by Montreal Cognitive Function Assessment Scale (MoCA) [25], and complete ability to perform activities of daily living assessed by Barthel index (BI) were assessed in all participants [26]. CI were divided by severity: subjective CI (SubCI)—the presence of cognitive complaints and MoCA ≥ 26; mild CI (MCI)—MoCA < 26 and normal independence; dementia—MoCA < 26 with loss of independence [27,28].

To assess individual cognitive functions, methods and tests, that previously showed high sensitivity in CSVD, were used [29].

### 3.3. Molecular Genetic Testing

The level of expression of protein-coding genes was assessed as part of the inventive panel created by the manufacturer of the biotechnology products, NanoString Technologies (Washington, DC, USA), according to the technical specifications of the researchers. The panel included 58 inflammation-associated genes. To assess expression, genes with reproducible GWAS loci associated with CSVD, AH, and AD were selected (“GWAS Catalog” (https://www.ebi.ac.uk/gwas/home, accessed on 4 June 2024) as well as genes whose circulating protein products associated with inflammation and BBB damage have demonstrated their role in the pathogenesis of CSVD [19,30,31,32,33]. The studied genes are listed in Appendix B (Table A2).

The differential gene expression was tested as part of the inventive panel by direct digital RNA detection using the NanoString nCounter platform (NanoString Technologies, Waltham, MA, USA). Total RNA was isolated from the leukocyte fraction of peripheral blood cells using the MagMax mirVana Total RNA Isolation Kit (Thermo Fisher Scientific Inc., Waltham, MA, USA) according to the manufacturer’s protocol. After hybridization of the probes and immobilization on a cartridge, the RNA samples were placed in the digital analyzer “nCounter Analysis System”. Processing of the obtained “raw” data, quality control, normalization, and analysis of differential gene expression (CSVD vs. healthy volunteers, CSVD MRI type 1 vs. healthy volunteers, and CSVD MRI type 2 vs. healthy volunteers) were performed using the nSolver4.0 software. Normalization was performed on four “reference” genes selected by the biotechnology company NanoString Technologies (Washington, DC, USA): alanyl-tRNA synthetase (*AARS*), ankyrin repeat and SOCS box containing 7 (*ASB7*), coiled-coil domain containing 127 (*CCDC127*), and CCR4-NOT transcription complex subunit 10 (*CNOT10*).

The differential gene expression was evaluated by determining the binary logarithm of the ratio of the values obtained in the groups (log2FC (fold change)), and the change in gene expression at |log2FC| > 1 was considered significant.

The results of differential gene expression obtained using the NanoString nCounter analyzer was confirmed by reverse transcription followed by quantitative real-time PCR for the same participants in the main and control groups. To confirm the differential expression, protein-coding genes were used, which showed significant changes in the expression level for the entire CSVD and MRI type 2 CSVD groups compared to the control group (acyl-CoA oxidase 1 (*ACOX1*), bridging integrator 1 (*BIN1*), CD2-associated protein (*CD2AP*), TNF receptor superfamily member 1A (*TNFR1*), and vascular endothelial growth factor A (*VEGFA*)). A comparative analysis of expression was carried out for two control genes, i.e., *AARS* and *ASB7*, from the reference genes used in NanoString. The inventive oligonucleotide primers were developed using the Vector NTI program and synthesized by ThermoFS (USA). Primers are listed in Appendix C (Table A3).

The obtained results were analyzed using the CFX Manager BioRad software (version 3.1) (calculation of the relative expression level). The level of mRNA expression was assessed by indicator C(t), i.e., threshold cycle reaction for the corresponding mRNA. The expression level of the tested genes was calculated by the ΔΔCt method.

### 3.4. Statistical Processing

The statistical analysis was carried out using the “IBM SPSS Statistics, 26.0” (IBM) software and in the programming language R version 4.2.2 (“RStudio”, version 2022.12.0-353).

Descriptive statistics included frequency and proportion (%) for categorical and ordinal variables and median, 1st, and 3rd quartiles or mean and standard deviation for quantitative variables. Two-sided versions of statistical criteria were used. The null hypothesis was rejected at *p* < 0.05. A comparative analysis of qualitative variables was carried out using the Pearson criterion χ2. The correlation analysis of quantitative variables was carried out using the Pearson method, with an assessment of the significance of the correlation. Quantitative variables were compared by the Mann–Whitney U test or Kruskal–Wallis H test. For post hoc pairwise comparisons, the Mann–Whitney U test was used, followed by Bonferroni correction for the multiple comparisons. The predictive value of changes in gene expression in relation to the development of a trait was assessed by ROC analysis.

The relative expression level of the tested genes was visualized by constructing a “heat map” in “RStudio” and the “gplots” package; hierarchical clustering was carried out using the “ward.D2” method at Euclidean distances between points and visualized using dendrograms.

## 4. Discussion

This study aimed to evaluate the expression profile of inflammation-associated genes in CSVD and its two diagnostic MRI types at the stage of confluent WMH (Fazekas grade 3). Gene expression was evaluated using the inventive panel for the NanoString nCounter analyzer. The inventive panel included protein-coding genes for which the GWAS-significant loci associated with CSVD, AH, and AD were repeatedly identified (“GWAS Catalog” (https://www.ebi.ac.uk/gwas/home, accessed on 4 June 2024)) as well as genes encoding proteins of inflammation and BBB damage, which have demonstrated their role in CSVD pathogenesis. These genes were selected for the panel from those significant for CSVD and AD because the former is the main modifiable risk factor for the latter, and as shown in morphological studies, mixed forms of the two diseases dominate in older adults and the elderly [20,21,34,35]. Chronic inflammation and its associated processes are one of the pathogenetic mechanisms underlying both CSVD and AD [36,37,38]. Clarification of common mechanisms significant for the two diseases as well as the identification of potential biomarkers of comorbidity will make it possible to predict the course of the diseases and the severity of their clinical presentation.

The study showed an association of MRI type 2 CSVD with reduced expression of the *ACOX1*, *CD33*, *CD2AP*, *TNFR1*, and *VEGFC* genes compared to the control group. We did not find any differences in the expression level for MRI type 1 compared to the control group. The two MRI types differed in the level of expression of the *CD33* gene, which was reduced in the second type. The results can confirm that the inflammatory reactions play a specific role in the MRI type 2 CSVD, that was suggested in a previous study [19].

In a comparative analysis of gene expression in MRI type 2, we found a decrease in the expression of *ACOX1*, *BIN1*, *CD2AP*, *CD33*, *TNFR1*, *VEGFA*, and *VEGFC* genes in clinically significant CI (MCI and dementia) compared to the control group. Meanwhile, the reduced expression of the *ACOX1* and *VEGFA* genes showed a prognostic value in the risk of MCI and dementia. The known functions of these genes suggest the potential pathways and mechanisms through which reduced gene expression is realized in MRI type 2 CSVD and associated CI.

The *ACOX1* gene encodes acyl-CoA oxidase-1, which is an enzyme of the β-oxidation pathway of long-chain fatty acids. Previous studies have shown that *ACOX1* deficiency leads to impaired myelination of white matter due to accumulation of long-chain fatty acids, oxidative stress, and hyperproduction of reactive oxygen species [39,40,41,42], while genetic deficiency of *ACOX1* causes pseudo-neonatal adrenoleukodystrophy [39], characterized by severe pathology of the white matter due to inflammatory demyelination [39,41]. Thus, the previously established role of reduced *ACOX1* expression in the demyelination of white matter involving inflammatory reactions [39,41] as well as the revealed significant decrease in *ACOX1* expression in MRI type 2 and the ability to predict clinically significant CI indicates the potential role of these findings in the formation of WMH and the development of CI in this population.

*VEGFA* is another gene whose reduced expression was significantly different from the control group. Understanding of the role of the *VEGFA* gene to the development and progression of cerebrovascular pathology is varied and controversial. This is probably due to many factors, such as the condition of the endothelium, the stage of the disease, the related mechanisms, and others. Previous studies in patients with cerebrovascular pathology have established an increased blood expression of the *VEGFA* gene [43,44,45], which was considered by the authors as a compensatory increase for remodeling small cerebral vessels. The fact that these results are not consistent with ours may indicate that vascular remodeling with the development of arteriolosclerosis in MRI type 2 is not the leading factor. This is indirectly confirmed by the fact that lacunes were less common with this type, and in general, it had a milder course compared to MRI type 1, suggesting a lesser severity of arteriolosclerosis-related ischemic mechanisms. However, significant evidence has shown the association of VEGFA deficiency with AD due to the antagonism of accumulating amyloid-β (Aß) with *VEGFA*, inhibition of VEGF-A-induced migration of endothelial cells, angiogenesis, neurogenesis, and neuroplasticity [46,47]. These changes are common to CSVD and AD [48,49], and the role of MRI type 2 in the development of mixed forms of CSVD with AD cannot be disregarded. This is consistent with a significant decrease in gene expression of another vascular endothelial growth factor in MRI type 2, *VEGFC.* It is a factor of lymphangiogenesis that provides glymphatic and meningeal lymph clearance and inhibition of neuroinflammation [50,51]. This mechanism is being actively studied in AD, and the revealed reduced expression of *VEGFC* in MRI type 2 CSVD confirms the importance of dysfunction of the glymphatic and meningeal lymphatic systems and neuroinflammation in CSVD [52].

The role of MRI type 2 and inflammation in general to the development of mixed forms of CSVD with AD can also be confirmed by a significant decrease in *CD33* expression in comparison with MRI type 1. *CD33* is expressed in cells with phagocytic activity and modulates inflammatory and immune responses [53]. The role of *CD33* as a risk factor for AD was established and confirmed with repeated GWAS [54]. An increase in *CD33* expression was shown to disrupt the synthesis of inflammatory cytokines, leading to a decrease in TNF-α, interleukin-8, and interleukin-1β levels [55], while suppression of *CD33* expression is associated with activation of immune cells, increased TNF-α production, and inflammation [56]. Thus, the reduced expression of *CD33* in MRI type 2 relative to the control group confirms the importance of inflammation in this CSVD variant as well as the role of immune cells activation, increased cytokine production in small cerebral vessels, and brain damage.

The *BIN1*, *CD2AP*, *CD33*, and *VEGFA* genes are considered in the literature as risk factors for AD. Our identification of significant differences in their expression in MRI type 2 and control group indicates the common pathogenetic pathways for this type and neurodegenerative pathology.

*CD2AP* is a significant risk factor for asthma, established during repeated GWAS. *CD2AP* participates in the clathrin-mediated transport of Aß through the BBB. In addition, the CD2-associated protein encoded by this gene is a scaffold protein highly expressed in endothelial cells of cerebral vessels and supports intercellular connections of endothelial cells [57,58]. Animal models have established the key role of *CD2AP* in maintaining the BBB in AD [59]. Like in asthma, the role of increased BBB permeability in the pathogenesis of CSVD was proven [5]. We established that the reduced expression of *CD2AP* in MRI type 2 CSVD indicates its important role in increased BBB permeability in this variant of disease. Another mechanism of damage to cerebral vessels and brain matter may be impaired clearance of Aß [60].

Binding integrator 1 (*BIN1* gene) belongs to the family of amphiphysins (amphiphysin II), functions as an adapter protein, and is an established regulator of endocytosis [61]. *BIN1* is the second most important risk factor for AD [62]. *BIN1* has been shown to be associated with tau and amyloid pathology [63] and is involved in inflammatory reactions [64] and apoptosis [65]. We did not find in the available literature studies on the level of *BIN1* expression in CSVD. Based on the studied functions of *BIN1*, the reduced expression of *BIN1* in the MRI type 2 CSVD established in this study may be associated with the depletion of its ability to carry out Aß transcytosis from the brain through the BBB into the vascular lumen. Another potential mechanism by which altered *BIN1* expression may be involved in CSVD may be its established role in maintaining neuroinflammation and the associated pathways [63,64].

The association of reduced *BIN1* expression with demyelination processes is characteristic of CSVD due to ischemia/hypoxia in the area of stenosed small vessels and vasogenic edema due to the BBB damage [31,66,67], as has been established in multiple sclerosis [68].

Tumor necrosis factor receptor-1 (*TNFR1* gene) is a TNF-α receptor. *TNFR1*-mediated signaling is crucial for the regulation of inflammatory and apoptotic reactions [69]. Studies on the role of TNF-R1 in the development of CSVD and CI are inconclusive. Specifically, it was found that in patients with vascular and degenerative CI, the plasma level of TNF-R1 is increased compared to the control group [70,71,72] and is significantly associated with the presence of lacunes in CSVD [73]. However, the protective role of elevated TNF-R1 levels in chronic inflammatory conditions has been shown. TNF-α induces the secretion of soluble TNF-R1 into the systemic circulation, which inhibits inflammation [74]. Hyperproduction of soluble TNF-R1 can weaken TNF-induced inflammatory processes by acting as decoy receptors for circulating TNF-α [75]. These data were confirmed in animal models of cerebral infarction caused by occlusion of the middle cerebral artery [76,77]. It is likely that the reduced expression of *TNFR1* found in MRI type 2 CSVD is the result of chronic inflammation and depletion of TNF-R1 protective mechanisms. This is consistent with previous studies that established an increased blood level of TNF-α in MRI type 2 CSVD [19].

## 5. Conclusions

MRI type 2 CSVD is characterized by a decrease in the expression of the *ACOX1*, *CD33*, *CD2AP*, *TNFR1*, and *VEGFC* genes relative to the control group. Previous studies have shown that changes in the expression of these genes are associated with inflammatory demyelination of the white matter, inhibition of VEGF-A-induced endothelial cell migration, angiogenesis, neurogenesis, glymphatic system dysfunction, increased BBB permeability with impaired Aβ clearance, and the progression of neuroinflammation. In addition, there is a direct connection of some of the genes with AD, which indicates the significance of the identified changes in the development of this CSVD variant and its potential role in comorbidity with AD.

## Figures and Tables

**Table 1:** Comparative characteristics of risk factors and clinical and neuroimaging manifestations of F3-grade MRI types of WMH.

Test	MRI Type 1 F3 (1) (*n* = 17)	MRI Type 2 F3 (2) (*n* = 17)	Control (0)	*p*
			(*n* = 11)	
Age (mean ± SD)	56.4 ± 8.1	67.1 ± 6.3	57.3 ± 9.7	*p* = 0.001
				*p*_1–2_ = 0.002
				*p*_0–2_ = 0.027
Gender (*n*, %)				
Female	10 (58.8%)	2 (11.8%)	7 (63.6%)	*p* = 0.018
Male	7 (41.2%)	15 (88.2%)	4 (36.4%)	
Education	15 [12; 16.5]	14 [13; 18]	16.5 [15; 17]	*p* > 0.05
AG (*n*, %)	17 (100%)	17 (100%)	4 (36.4%)	*p* < 0.001
Hypertension degree:				
Degree 1	0 (0%)	1 (5.9%)	2 (18.2%)	
Degree 2	5 (29.4%)	6 (35.3%)	2 (18.2%)	
Degree 3	12 (70.6%)	10 (58.8%)	0 (0%)	
Type 2 diabetes (*n*, %)	3 (17.7%)	5 (29.4%)	-	*p* > 0.05
Smoking (*n*, %)	10 (58.8%)	7 (41.2%)	3 (27.3%)	*p* > 0.05
Obesity (*n*, %)	5 (29.4%)	10 (58.8%)	3 (27.3%)	*p* > 0.05
Cholesterol level, mmol/L (Me [Q25%; Q75%])	5.7 [4.2; 6.8]	5.6 [4.7; 5.9]	5.2 [4.8; 5.8]	*p* > 0.05
MoCA (Me [Q25%; Q75%])	21 [17; 23]	24 [21; 26]	28 [27; 29]	*p* < 0.001
				*p*_0–1_ < 0.001
				*p*_0–2_ = 0.006
Cognitive functions				
(Me [Q25%; Q75%]):				
Inhibition—Stroop test (s)	226 [211; 301]	150 [135; 206]	118 [102; 179]	*p* = 0.009
				*p*_0–1_ = 0.001
				*p*_1–2_ = 0.033
Switching—TMT				
(Trail making test) test B-A (s)	91 [45; 223]	115 [70; 171]	32 [19; 90]	*p* = 0.011
				*p*_0–1_ = 0.026
				*p*_0–2_ = 0.019
Productivity—Verbal fluency test (animals)	7 [4; 9]	11 [8; 13]	15 [14; 19]	*p* < 0.000
				*p*_0–1_ < 0.000
				*p*_0–2_ = 0.017
Memory—Delayed playback of 10 words (words)	3 [2; 6]	6 [5; 8]	9 [8;9]	*p* = 0.001
				*p*_0–1_ = 0.001
CI (*n*, %)	17 (100%)	17 (100%)	-	*p* > 0.05
Dementia	6 (35.3%)	5 (29.4%)		
MCI	10 (58.8%)	6 (35.3%)		
subCI	1 (5.9%)	6 (35.3%)		
MRI signs:				
Lacunes (*n*, %)	17 (100%)	9 (52.9%)	-	*p* = 0.018
Microbleeds (*n*, %)	17 (100%)	8 (47.1%)	-	*p* = 0.001
Atrophy (*n*, %)	16 (94.1%)	13 (76.5%)	-	*p* > 0.05
Enlarged PVS (*n*, %)	17 (100%)	17 (100%)	-	

Notes: DM—diabetes mellitus; MoCA—Montreal Cognitive Function Assessment Scale; CI—cognitive impairment; MCI—mild CI; SubCI—subjective CI; PVS—perivascular spaces.

## Data Availability

Raw data were generated at the Research Center of Neurology. The data that support the findings of this study are available from the corresponding author upon reasonable request. Clinical, neuroimaging, laboratory, and statistical data will be available upon request from any qualified investigator.

## Appendix A

**Table A1:** Neuroimaging and clinical and laboratory features of two MRI types of CSVD at the grade 3 Fazekas WMH.

F3 MRI Type 1	F3 MRI Type 2
**Neuroimaging characteristics**	

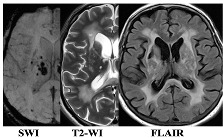
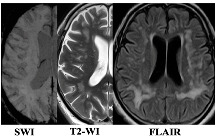
	
**WMH:** more pronounced in the periventricular and deep regions of the cerebral hemispheres, subcortical structures, external capsule, brainstem, and cerebellum	**WMH:** periventricular—in the posterior cerebral hemispheres, deep—in the frontal and parietal lobes
**Lacunes:** multiple in subcortical structures and cerebral white matter	**Lacunes:** single in the white matter of the cerebral hemispheres
**CMB:** juxtacortical CMHs in all regions of the cerebral hemispheres, in subcortical structures	**CMB:** single juxtacortical CMHs in the white matter of the temporal and parietal lobes
Cerebral atrophy: more pronounced	**Cerebral atrophy:** less pronounced
**Enlarged PVS:** pronounced in subcortical structures	**Enlarged PVS:** extended
**Clinical characteristics**	
**Cognitive impairment:** more pronounced (MCI and dementia)	**Cognitive impairment:** less pronounced (SubCI and MCI)
**Gait disorders:** more pronounced	**Gait disorders:** less pronounced
**Laboratory testing**	
Reduced VEGF-A	Increased TNF-α

Notes: SWI—susceptibility-weighted imaging; T2-WI—T2-weighted image; FLAIR—fluid-attenuated inversion recovery; WMH—white matter hyperintensities; CMB—cerebral microbleeds; PVS—perivascular spaces; MCI—mild cognitive impairment; SubCI—subjective cognitive impairment; VEGF-A—vascular endothelial growth factor A; TNF-α—tumor necrosis factor-α.

## Appendix B

**Table A2:** A list of the tested genes and their corresponding proteins.

No.	Gene	Protein
GWAS in CSVD		
1	*ACOX1*	acyl-CoA oxidase 1
2	FBN2	fibrillin 2
3	APOE	apolipoprotein E
4	LINC00539	long intergenic non-protein coding RNA 539
5	COL4A2	collagen type IV alpha 2 chain
6	FBF1	Fas binding factor 1
7	MRPL38	mitochondrial ribosomal protein L38
8	NOS3	nitric oxide synthase 3
9	PMF1	polyamine modulated factor 1
10	SLC25A44	solute carrier family 25 member 44
11	TRIM65	tripartite motif containing 65
12	TRIM47	tripartite motif containing 47
13	*WBP2*	WW domain binding protein 2
14	*PLEKHG1*	pleckstrin homology and RhoGEF domain containing G1
15	*CST3*	cystatin C
16	*FRMD1*	FERM domain containing 1
GWAS in AD		
17	*BIN1*	bridging integrator 1
18	*CD33*	CD33 molecule
19	*CD2AP*	CD2-associated protein
20	*ABCA7*	ATP binding cassette subfamily A member 7
21	*CH25H*	cholesterol 25-hydroxylase
22	*CLU*	clusterin
23	*CR1*	complement receptor 1
24	*EPHA1*	EPH receptor A1
25	*PICALM*	phosphatidylinositol binding clathrin assembly protein
26	*MS4A6A*	membrane spanning 4-domains A6A
27	*SORL1*	Sortilin-related receptor 1
28	*TREM2*	triggering receptor expressed on myeloid cells 2
Hypertension risk genes		
29	*AGT*	angiotensinogen
30	*ACE*	angiotensin converting enzyme
31	*CYP11B2*	cytochrome P450 family 11 subfamily B member 2
32	AGTR1	angiotensin II receptor type 1
33	NPPB	natriuretic peptide B
Circulating blood markers in CSVD: Functioning of the BBB		
34	*AQP1*	aquaporin 1
35	*CA4*	carbonic anhydrase 4
36	*CLDN5*	claudin 5
37	*MMP2*	matrix metallopeptidase 2
38	*MMP3*	matrix metallopeptidase 3
39	*MMP9*	matrix metallopeptidase 9
Circulating blood markers in CSVD: Systemic inflammation		
40	*CRP*	C-reactive protein
41	*FGB*	fibrinogen beta chain
42	*FGA*	fibrinogen alpha chain
43	*FGG*	fibrinogen gamma chain
44	*IL1*	interleukin-1
45	*IL1B*	interleukin-1B
46	*IL6*	interleukin-6
47	*TNF*	tumor necrosis factor
48	TNFR1	TNF receptor superfamily member 1A
49	TNFR2	TNF receptor superfamily member 1B
Circulating blood markers in CSVD: Local inflammation/endothelial dysfunction		
50	VCAM1	vascular cell adhesion molecule 1
51	ICAM1	intercellular adhesion molecule 1
52	MTHFR	methylenetetrahydrofolate reductase
53	PSEL	selectin P
54	PLAT	tissue-type plasminogen activator
Circulating blood markers in CSVD: Trophic factors		
55	BDNF	Brain-derived neurotrophic factor
56	TGFB1	transforming growth factor beta 1
57	VEGFA	vascular endothelial growth factor A
58	*VEGFC*	vascular endothelial growth factor C
Reference genes		
59	*AARS*	alanyl-tRNA synthetase
60	*ASB7*	ankyrin repeat and SOCS box containing 7
61	*CCDC127*	coiled-coil domain containing 127
62	*CNOT10*	CCR4-NOT transcription complex subunit 10

## Appendix C

**Table A3:** Sequences of specific oligonucleotide primers and fluorescent probes for the tested and reference genes.

Oligonucleotide	Oligonucleotide Sequence	Label, 5′	Label, 3′
	5′ → 3′		
AARS1	F: GATTACTTTAAGGAATTGGCATGTAA		
	R: CAATGGGAATGCCAAACTCT		
	P: GGCTCTGGAACTCCTCACCC	FAM	BHQ1
ASB7	F: GCATCGTCTGTAAAGGTTAAGAAG		
	R: GGGAGGGGCTTCAAAGTA		
	P: TTTTTGCTCTGTAGCCTGGACG	FAM	BHQ1
ACOX1	F: CTGGTGGGCTTGGAAAGA		
	R: TTTCCCCTTAGTGATGAGCTG		
	P: TTCAAATCATGCAATAGTTCTTGCC	FAM	BHQ1
BIN1	F: TCCGTCAAAGCCATGCAC		
	R: CCAATCGGGCTCATACACCT		
	P: GAGGCTTCCAAGAAGCTGAATGA	FAM	BHQ1
CD2AP	F: AGGACGATTCAGAAACTGTTTTG		
	R: CCAGATGCAGTTTCACTCACA		
	P: GCTGGGCCTACTTCACCTATACC	FAM	BHQ1
TNFR1	F: CCTGAAAAAGAGGGGGAGCT		
	R: GTGAAGCCTGGAGTGGGACT		
	P: CTACTAAGCCCCTGGCCCCA	FAM	BHQ1
VEGFA	F: GAATCATCACGAAGTGGTGAAG		
	R: AAGATGTCCACCAGGGTCTC		
	P: TCAGCGCAGCTACTGCCATC	FAM	BHQ1

Abbreviations: F—forward primer; R—reverse primer; P—probe; FAM—fluorescent label; BHQ1—quencher molecule.
